# Assessment of risk for pre‐eclampsia at mid‐gestation to define subsequent care

**DOI:** 10.1002/uog.29222

**Published:** 2025-04-18

**Authors:** S. Adjahou, V. Logdanidis, A. Wright, A. Syngelaki, R. Akolekar, K. H. Nicolaides

**Affiliations:** ^1^ Fetal Medicine Research Institute, King's College Hospital London UK; ^2^ Institute of Health Research University of Exeter Exeter UK; ^3^ Fetal Medicine Unit, Medway Maritime Hospital, Gillingham UK; ^4^ Institute of Medical Sciences Canterbury Christ Church University Chatham UK

**Keywords:** estimated fetal weight, fetal biometry, impaired placentation, mean arterial pressure, pre‐eclampsia, uterine artery Doppler

## Abstract

**Objective:**

To stratify pregnancy care based on the estimated risk of pre‐eclampsia (PE) from screening at 19–24 weeks' gestation by combinations of maternal risk factors, estimated fetal weight (EFW), mean arterial pressure (MAP) and uterine artery pulsatility index (UtA‐PI).

**Methods:**

The data for this study were derived from a prospective non‐interventional study in 134 443 women with a singleton pregnancy attending for a routine ultrasound scan at 19 + 0 to 23 + 6 weeks' gestation in two UK maternity hospitals. The visit included recording of maternal demographic characteristics and medical history, sonographic EFW and measurement of MAP and UtA‐PI. The competing‐risks model was used to estimate the individual patient‐specific risk of delivery with PE at < 28, < 32 and < 36 weeks' gestation. Receiver‐operating‐characteristics curves were constructed for screen‐positive rates (SPRs) at different detection rates of delivery with PE at < 28, < 32 and < 36 weeks' gestation for the combinations of maternal risk factors, EFW and MAP, and of maternal risk factors, EFW, MAP and UtA‐PI. Different risk cut‐offs were used with the intention of detecting about 80%, 85% and 90% of cases of delivery with PE at < 28, < 32 and < 36 weeks' gestation. Calibration for risk of delivery with PE at < 28, < 32 and < 36 weeks' gestation was assessed by plotting the observed incidence of PE against the predicted incidence of PE.

**Results:**

The study population contained 4335 (3.2%) women that subsequently developed PE, including 64 (0.05%) that delivered with PE at < 28 weeks' gestation, 209 (0.2%) that delivered with PE at < 32 weeks and 655 (0.5%) that delivered with PE at < 36 weeks. If the objective of screening was to identify about 90% of cases of delivery with PE at < 28, < 32 and < 36 weeks and the method of screening was a combination of maternal risk factors, EFW and MAP, the respective SPRs would be 11.0%, 18.3% and 38.8%. If the method of screening also included UtA‐PI, the respective SPRs would be 2.6%, 3.8% and 23.6%. If the objective of screening was to identify about 80% of cases of delivery with PE at < 28, < 32 and < 36 weeks and the method of screening was a combination of maternal risk factors, EFW and MAP, the respective SPRs would be 5.9%, 9.7% and 21.9%. If the method of screening also included UtA‐PI, the respective SPRs would be 1.0%, 2.1% and 11.7%. The calibration plots demonstrated good agreement between the estimated risk and observed incidence of PE.

**Conclusions:**

All women should be offered assessment of risk for PE at 11–13 weeks, to help identify those requiring aspirin prophylaxis to reduce the rate of preterm PE, and at 35–37 weeks, to determine the optimal timing of birth to reduce the rate of term PE. Assessment of risk for PE at mid‐gestation can be used to identify the subgroups that require additional monitoring at 24–35, 28–35 and 32–35 weeks' gestation. The best performance of screening, reflected in the SPR necessary to achieve a high detection rate, is achieved by a combination of maternal risk factors, MAP and UtA‐PI. © 2025 The Author(s). *Ultrasound in Obstetrics & Gynecology* published by John Wiley & Sons Ltd on behalf of International Society of Ultrasound in Obstetrics and Gynecology.

## INTRODUCTION

Pre‐eclampsia (PE) complicates about 5% of all pregnancies and is a major cause of short‐ and long‐term mortality and morbidity for the mother and fetus/neonate^1^. Effective prediction of delivery with PE can be achieved using the competing‐risks model, which combines the prior risk, based on maternal demographic characteristics and elements from the medical history, with biophysical and biochemical measurements in pregnancy^2^, ^3^, ^4^, ^5^. Prevention of PE can be achieved by a two‐stage strategy. First, assessment of risk for preterm PE at 12 weeks' gestation, which identifies about 75% of cases at a 10% screen‐positive rate (SPR)^3^, and treatment of the high‐risk group with aspirin (150 mg/day) from 12 to 36 weeks' gestation, which results in a consequent reduction of preterm PE by about 60%^6^. Second, assessment of risk for subsequent PE at 36 weeks' gestation, which identifies about 75% of cases at a 10% SPR^5^, and appropriate timing of birth to reduce the rate of term PE by about 30%^7^.

We have proposed previously that, in addition to assessment of PE risk at 12 and 36 weeks' gestation, screening for PE should also be carried out at around 20 weeks^8^, ^9^. The rationale for such mid‐gestation screening is the identification of a high‐risk group that would benefit from close monitoring at 24–36 weeks' gestation to minimize adverse perinatal events for those that develop PE by determining the appropriate timing and place for delivery. Useful biomarkers for PE at 20 weeks' gestation that could easily be assessed during a routine ultrasound examination are estimated fetal weight (EFW)^10^, mean arterial pressure (MAP) and uterine artery pulsatility index (UtA‐PI)^8^, ^9^, ^11^.

The objective of this study of 134 443 women with a singleton pregnancy attending for a routine ultrasound scan at 19 + 0 to 23 + 6 weeks' gestation was to stratify pregnancy care based on the estimated risk of delivery with PE at < 28, < 32 and < 36 weeks' gestation by a combination of maternal risk factors with EFW, MAP and UtA‐PI. Different risk cut‐offs were used with the intention of detecting about 80%, 85% and 90% of cases of delivery with PE at < 28, < 32 and < 36 weeks. The rationale for such approach is that those at high‐risk for delivering with PE at < 28 weeks would require close monitoring for high blood pressure and proteinuria at 24–35 weeks, those at high risk for PE at < 32 weeks would require monitoring for high blood pressure and proteinuria at 28–35 weeks, and those at high‐risk for PE at < 36 weeks would require close monitoring for high blood pressure and proteinuria at 32–35 weeks.

## METHODS

### Study design and participants

The data for this study were derived from prospective screening for adverse obstetric outcome in women attending for routine pregnancy care at 19 + 0 to 23 + 6 weeks' gestation at King's College Hospital, London, and Medway Maritime Hospital, Gillingham, UK, between January 2011 and May 2024. At this visit, first, we recorded maternal demographic characteristics and medical history; second, we carried out an ultrasound examination of fetal anatomy and measurement of fetal head circumference, abdominal circumference and femur length to calculate the EFW using the Hadlock formula^12^, identified as the most accurate model in a systematic review^13^; third, we measured MAP using validated automated devices according to a standardized protocol^14^; and fourth, we measured left and right UtA‐PI using either transvaginal or transabdominal color Doppler ultrasound and calculated the mean value of the two arteries^15^, ^16^. The majority of UtA‐PI measurements were carried out transvaginally because at the same time we were measuring cervical length; the transabdominal approach was used when women declined transvaginal sonography. Ultrasound scans were carried out by sonographers with extensive training in ultrasound scanning who had obtained the appropriate Fetal Medicine Foundation certificate of competence in ultrasound and Doppler examinations. Gestational age was determined from measurement of fetal crown–rump length at 11–13 weeks^17^.

Women gave written informed consent to participate in the study, which was approved by the National Health Service Research Ethics Committee (No. 02‐03‐033). The inclusion criteria for this study were singleton pregnancy resulting in a phenotypically normal live birth or stillbirth at ≥ 24 weeks' gestation. We excluded pregnancies with known aneuploidy or major fetal abnormality and those resulting in a miscarriage or termination of pregnancy.

### Outcome measures

Outcome measures were delivery with PE at < 28, < 32 and < 36 weeks' gestation. Data on pregnancy outcome were collected from the hospital maternity records or the general medical practitioners of the women. The obstetric records of all women with chronic hypertension or pregnancy‐associated hypertension were examined to determine the diagnosis of PE. This was based on the finding of new‐onset hypertension (systolic blood pressure of ≥ 140 mmHg or diastolic blood pressure of ≥ 90 mmHg on at least two occasions 4 h apart developing after 20 weeks' gestation in previously normotensive women) or chronic hypertension and at least one of the following: proteinuria (≥ 300 mg/24 h or protein‐to‐creatinine ratio ≥ 30 mg/mmoL or ≥ 2+ on dipstick testing), renal insufficiency with serum creatinine > 97 μmol/L in the absence of underlying renal disease, hepatic dysfunction with blood concentration of transaminases more than twice the upper limit of normal (≥ 65 IU/L for our laboratory), thrombocytopenia (platelet count < 100 000/μL), neurological complications (e.g. cerebral or visual symptoms) or pulmonary edema^18^.

### Statistical analysis

Data were expressed as median (interquartile range (IQR)) for continuous variables and *n* (%) for categorical variables. Student's *t*‐test and chi‐square test or Fisher's exact test were used for comparing outcome groups for continuous and categorical data, respectively. *P* < 0.05 was considered statistically significant.

Fetal weight was estimated using the Hadlock 3 formula^12^, and the Fetal Medicine Foundation fetal and neonatal population‐weight charts were used to calculate EFW *Z*‐scores^19^. A scatterplot of EFW *Z*‐score against gestational age at delivery with PE was produced. The relationship between EFW *Z*‐score and gestational age at delivery with PE was assumed to be linear, up until the mean EFW *Z*‐score of zero. Beyond this point, and for unaffected pregnancies, the mean was assumed to be zero. Correlations between the EFW *Z*‐score and multiples of the median (MoM) values of MAP and UtA‐PI were also calculated.

The competing‐risks model was used to estimate the individual patient‐specific risk of delivery with PE at < 28, < 32 and < 36 weeks' gestation by a combination of maternal demographic characteristics and medical history with biomarkers^2^. The competing‐risks approach is based on a survival‐time model for gestational age at delivery with PE. In our approach, we assumed that, if the pregnancy was to continue indefinitely, all women would develop PE and whether they do so or not before a specified gestational age depends on competition between delivery before or after development of PE. The posterior distribution of gestational age at delivery with PE was obtained using Bayes' theorem by multiplying the prior probability density from maternal factors by the likelihood function from biomarker MoM values^20^, ^21^ and *Z*‐scores. The measured values of biomarkers were converted to MoMs or *Z*‐scores to remove the effects of characteristics such as gestational age, maternal weight, race, method of conception, medical conditions and elements from the obstetric history of the individual.

Detection rates (DRs) were plotted against SPRs for delivery with PE at < 28, < 32 and < 36 weeks' gestation for the combinations of maternal risk factors, EFW and MAP, and of maternal risk factors, EFW, MAP and UtA‐PI. Different risk cut‐offs were used with the intention of detecting about 80%, 85% and 90% of cases of delivery with PE at < 28, < 32 and < 36 weeks' gestation.

Calibration for risk of delivery with PE at < 28, < 32 and < 36 weeks' gestation was assessed by plotting the observed incidence of PE against the predicted incidence of PE. The plots were produced by grouping the data into bins according to risk. Calibration‐in‐the‐large is a measure of whether the risk is generally too high or too low. To quantify this, logistic regression models were fitted using delivery with PE at < 36 weeks as the outcome and the logit risk as a predictor. First, we estimated the intercept from a logistic regression of incidence on the logit of risk with the slope fixed at 1. If there is a general tendency for underestimation, such that the observed incidence is larger than the predicted incidence, the intercept will be positive. Conversely, if there is a tendency for overestimation, the intercept will be negative. Second, we refitted the model for the slope to assess the calibration across the range of risks. If the risk is well‐calibrated, the slope will be 1.0.

The statistical software package R was used for data analysis^22^.

## RESULTS

### Study participants

The study population of 134 443 women with a singleton pregnancy included 4335 (3.2%) women that subsequently developed PE, including 64 (0.05%) that delivered with PE at < 28 weeks' gestation, 209 (0.2%) that delivered with PE at < 32 weeks and 655 (0.5%) that delivered with PE at < 36 weeks. The characteristics of the study population are summarized in Table 1. In the PE group, compared with unaffected pregnancies, there was a higher median maternal age, weight and body mass index, higher incidence of women of black race, those with a history of chronic hypertension and diabetes mellitus, family history of PE, conception by *in‐vitro* fertilization, nulliparous women and parous women with a history of PE, longer median interpregnancy interval and lower incidence of smokers.

**Table 1:** Maternal and pregnancy characteristics of study population of 134 443 pregnancies, according to development of pre‐eclampsia (PE) and gestational age (GA) at delivery with PE

Characteristic	No PE (*n* = 130 108)	All PE (*n* = 4335)	*P* *	PE with delivery < 36 weeks* (*n* = 655)	*P* *	PE with delivery < 32 weeks* (*n* = 209)	*P* *
Age (years)	31.7 (27.5–35.3)	32.0 (27.6–35.9)	0.001	31.8 (27.6–35.9)	0.185	31.7 (27.7–36.4)	0.077
Weight (kg)	68.0 (60.0–79.0)	74.3 (64.0–89.1)	< 0.0001	75.0 (65.0–88.3)	< 0.0001	75.0 (65.8–88.0)	< 0.0001
Height (cm)	165 (161–170)	165 (160–169)	0.021	164 (160–168)	< 0.0001	164 (159–168)	< 0.0001
BMI (kg/m^2^)	24.8 (22.1–28.9)	27.3 (23.7–32.7)	< 0.0001	27.6 (24.2–32.9)	< 0.0001	28.4 (24.5–32.8)	< 0.0001
GA (weeks)	21.6 (21.0–22.0)	21.6 (21.0–22.0)	0.139	21.6 (21.0–22.1)	0.076	21.6 (21.1–22.1)	0.028
Race			< 0.0001		< 0.0001		< 0.0001
White	96 462 (74.14)	2843 (65.58)		355 (54.20)		93 (44.50)	
Black	20 082 (15.43)	1140 (26.30)		234 (35.73)		95 (45.45)	
South Asian	6971 (5.36)	192 (4.43)		43 (6.56)		16 (7.66)	
East Asian	2616 (2.01)	62 (1.43)		7 (1.07)		2 (0.96)	
Mixed	3977 (3.06)	98 (2.26)		16 (2.44)		3 (1.44)	
Medical history							
CH	1195 (0.92)	468 (10.80)	< 0.0001	109 (16.64)	< 0.0001	36 (17.22)	< 0.0001
DM Type I	580 (0.45)	64 (1.48)	< 0.0001	26 (3.97)	< 0.0001	4 (1.91)	< 0.0001
DM Type II	857 (0.66)	82 (1.89)	< 0.0001	27 (4.12)	< 0.0001	9 (4.31)	< 0.0001
SLE/APS	328 (0.25)	13 (0.30)	0.644	3 (0.46)	0.512	2 (0.96)	0.181
Smoker	9563 (7.35)	175 (4.04)	< 0.0001	25 (3.82)	0.0007	7 (3.35)	0.037
Family history of PE	4659 (3.58)	317 (7.31)	< 0.0001	53 (8.09)	< 0.0001	19 (9.09)	< 0.0001
Method of conception			< 0.0001		< 0.0001		0.322
Natural	124 708 (95.85)	4011 (92.53)		602 (91.91)		196 (93.78)	
*In‐vitro* fertilization	4632 (3.56)	290 (6.69)		44 (6.72)		11 (5.26)	
Ovulation drugs	768 (0.59)	34 (0.78)		9 (1.37)		2 (0.96)	
Parity							
Nulliparous	59 317 (45.59)	2684 (61.91)	< 0.0001	397 (60.61)	< 0.0001	123 (58.85)	< 0.0001
Parous, no previous PE	67 513 (51.89)	1139 (26.27)	< 0.0001	142 (21.68)	< 0.0001	48 (22.97)	< 0.0001
Parous, previous PE	3278 (2.52)	512 (11.81)	< 0.0001	116 (17.71)	< 0.0001	38 (18.18)	< 0.0001
Interpregnancy interval (years)	2.8 (1.7–4.6)	3.5 (2.1–6.2)	< 0.0001	3.6 (2.2–7.0)	< 0.0001	3.7 (2.0–6.9)	0.046

Data are given as median (interquartile range) or *n* (%).

* Comparison with no‐PE group. APS, antiphospholipid syndrome; BMI, body mass index; CH, chronic hypertension; DM, diabetes mellitus; SLE, systemic lupus erythematosus.

### Distribution of biomarkers in pregnancies with PE

**Figure 1:**
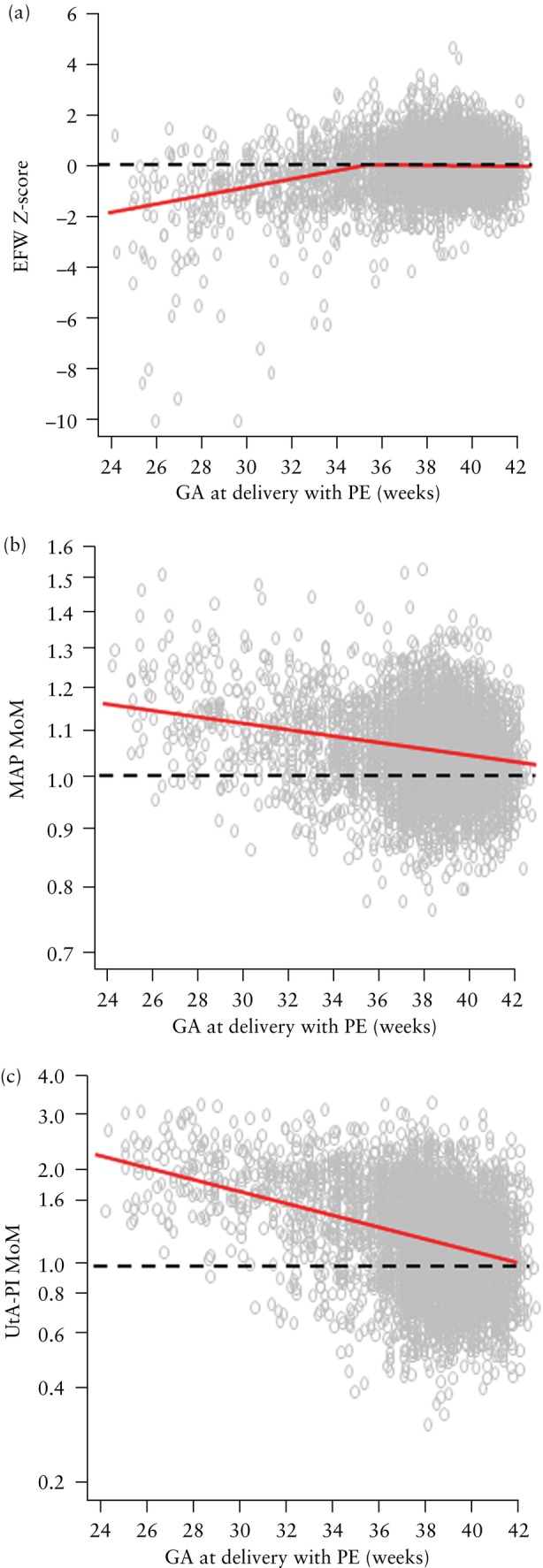
Estimated fetal weight (EFW) *Z*‐score (a), mean arterial pressure (MAP) multiples of the median (MoM) (b) and uterine artery pulsatility index (UtA‐PI) MoM (c) at 19 + 0 to 23 + 6 weeks' gestation, plotted against gestational age (GA) at delivery with pre‐eclampsia (PE). Red lines are fitted relationships.

The distribution of EFW *Z*‐score according to gestational age at delivery with PE is illustrated in Figure 1a. The EFW *Z*‐score was low in pregnancies delivering with PE and increased with gestational age at delivery (intercept, −9.7012 (95% CI, −10.6407 to −6.2003); slope, 0.2812 (95% CI, 0.1723–0.3094)). The average EFW *Z*‐score for those pregnancies delivering with PE after 34 + 4 weeks is assumed to be zero. The incidence of a small‐for‐gestational‐age (SGA) neonate with birth weight < 10^th^ percentile was 89.1% (57/64) for pregnancies with PE delivering at < 28 weeks' gestation, 82.1% (119/145) for pregnancies with PE delivering between 28 + 0 and 31 + 6 weeks, 66.8% (298/446) for pregnancies with PE delivering between 32 + 0 and 35 + 6 weeks and 22.5% (827/3680) for pregnancies with PE delivering at ≥ 36 weeks.

The distribution of log_10_ MAP MoM according to gestational age at delivery with PE is illustrated in Figure 1b. The log_10_ MAP MoM was high in pregnancies delivering with PE and decreased with gestational age at delivery (intercept, 0.1227 (95% CI, 0.1064–0.1382); slope, −0.002662 (95% CI, −0.003075 to −0.002235)). The distribution of log_10_ UtA‐PI MoM according to gestational age at delivery with PE is illustrated in Figure 1c. The log_10_ UtA‐PI MoM was high in pregnancies delivering with PE and decreased with gestational age at delivery (intercept, 0.9262 (95% CI, 0.8629–0.9956); slope, −0.02276 (95% CI, −0.02464 to −0.02108)).

The correlation coefficient was −0.007 (95% CI, −0.013 to −0.002) between EFW *Z*‐score and log_10_ MAP MoM, −0.062 (95% CI, −0.068 to −0.057) between EFW *Z*‐score and log_10_ UtA‐PI MoM, and −0.041 (95% CI, −0.047 to −0.035) between log_10_ MAP MoM and log_10_ UtA‐PI MoM.

### Prediction of delivery with PE at < 28, < 32 and < 36 weeks

**Figure 2:**
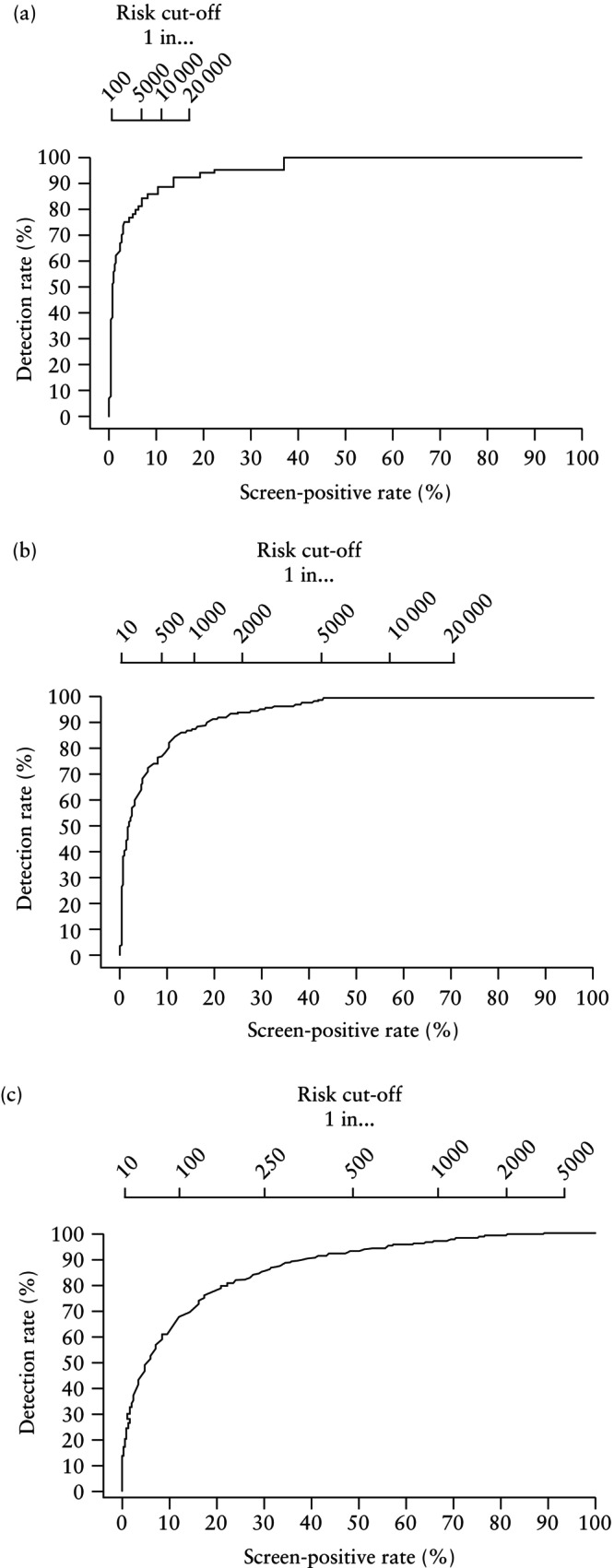
Receiver‐operating‐characteristics curves for prediction of delivery with pre‐eclampsia at (a) < 28 weeks, (b) < 32 weeks and (c) < 36 weeks' gestation, in screening by a combination of maternal risk factors with estimated fetal weight and mean arterial pressure. On top of each panel are the risk cut‐offs (i.e. 1 in…).

**Figure 3:**
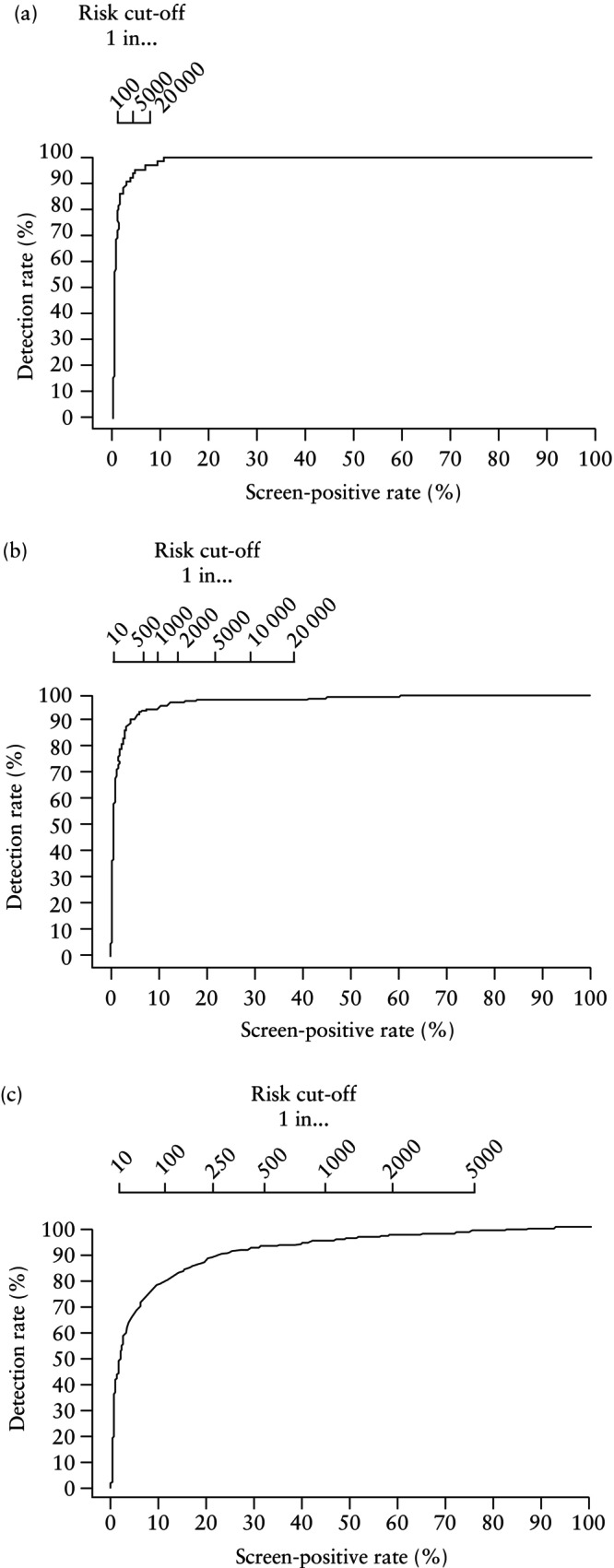
Receiver‐operating‐characteristics curves for prediction of delivery with pre‐eclampsia at (a) < 28 weeks, (b)< 32 weeks and (c) < 36 weeks' gestation, in screening by a combination of maternal risk factors with estimated fetal weight, mean arterial pressure and uterine artery pulsatility index. On top of each panel are the risk cut‐offs (i.e. 1 in…).

The performance of screening for delivery with PE at < 28, < 32 and < 36 weeks' gestation by a combination of, first, maternal risk factors, EFW and MAP, and second, maternal risk factors, EFW, MAP and UtA‐PI, is illustrated in Figures 2 and 3, respectively. Risk cut‐offs and SPRs in screening with the intention of detecting about 80%, 85% and 90% of cases of delivery with PE at < 28, < 32 and < 36 weeks' gestation are shown in Tables S1–S3 and summarized in Table 2.

**Table 2:** Prediction of delivery with pre‐eclampsia (PE) at < 28, < 32 and < 36 weeks' gestation: risk cut‐off and screen‐positive rate (SPR) to achieve detection rates of 80%, 85% and 90% in screening at mid‐gestation by maternal risk factors, estimated fetal weight (EFW) and mean arterial pressure (MAP), and by maternal risk factors, EFW, MAP and uterine artery pulsatility index (UtA‐PI)

	Maternal risk factors, EFW and MAP		Maternal risk factors, EFW, MAP and UtA‐PI	
Desired detection rate	Risk cut‐off	SPR (% (95% CI))	Risk cut‐off	SPR (% (95% CI))
Delivery with PE < 28 weeks				
80%	1 in 4000	5.9 (5.8–6.0)	1 in 300	1.0 (1.0–1.1)
85%	1 in 6000	7.9 (7.7–8.0)	1 in 600	1.4 (1.4–1.5)
90%	1 in 10 000	11.0 (10.8–11.1)	1 in 2000	2.6 (2.5–2.7)
Delivery with PE < 32 weeks				
80%	1 in 570	9.7 (9.5–9.8)	1 in 75	2.1 (2.0–2.1)
85%	1 in 700	11.5 (11.4–11.7)	1 in 117	2.7 (2.6–2.8)
90%	1 in 1250	18.3 (18.1–18.5)	1 in 200	3.8 (3.7–3.9)
Delivery with PE < 36 weeks				
80%	1 in 175	21.9 (21.7–22.2)	1 in 110	11.7 (11.6–11.9)
85%	1 in 240	29.2 (29.0–29.5)	1 in 180	16.7 (16.5–16.9)
90%	1 in 350	38.8 (38.6–39.1)	1 in 300	23.6 (23.4–23.8)

If the objective of screening was to identify about 90% of cases of delivery with PE at < 28, < 32 and < 36 weeks and the method of screening was a combination of maternal risk factors, EFW and MAP, the respective SPRs would be 11.0%, 18.3% and 38.8%. If the method of screening also included UtA‐PI, the respective SPRs would be 2.6%, 3.8% and 23.6%. If the objective of screening was to identify about 80% of cases of delivery with PE at < 28, < 32 and < 36 weeks and the method of screening was a combination of maternal factors, EFW and MAP, the respective SPRs would be 5.9%, 9.7% and 21.9%. If the method of screening also included UtA‐PI, the respective SPRs would be 1.0%, 2.1% and 11.7%.

### Validation of predictive models for delivery with PE

**Figure 4:**
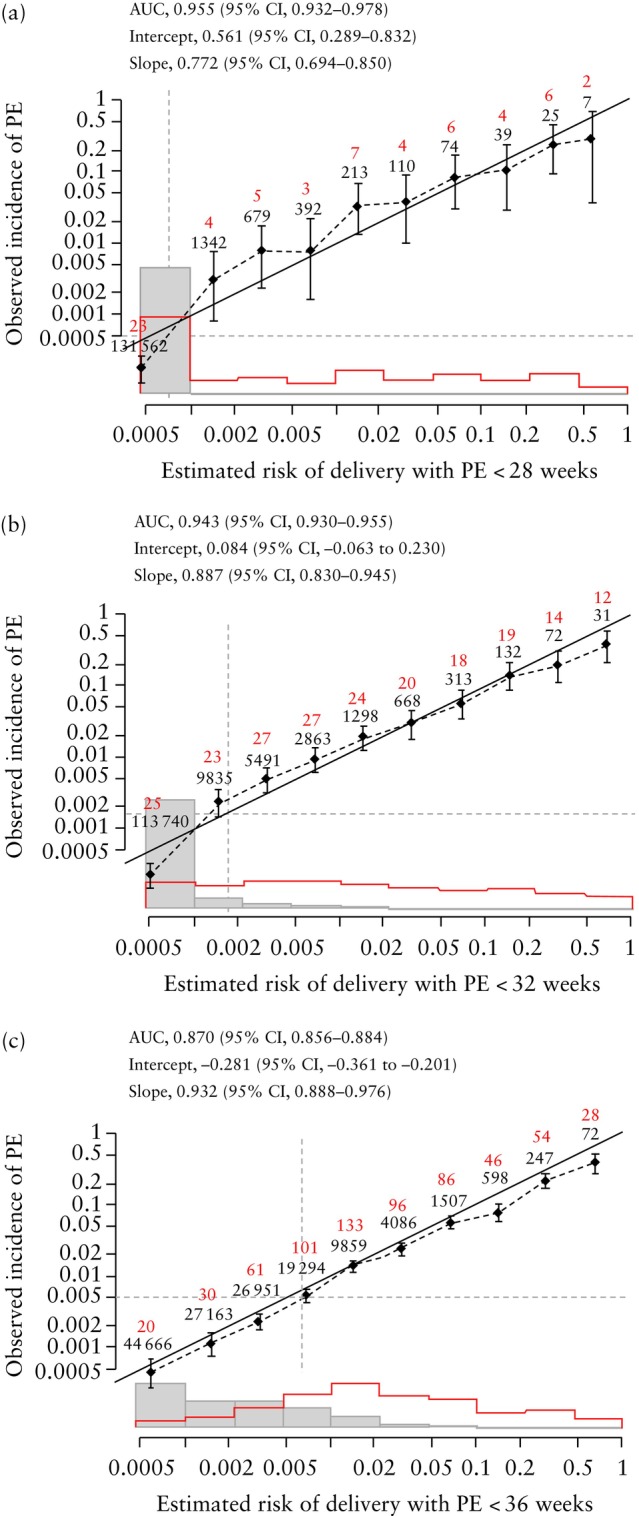
Calibration plots for estimated risk of delivery with pre‐eclampsia (PE) at (a) < 28 weeks, (b) < 32 weeks and (c) < 36 weeks' gestation in screening by maternal risk factors, estimated fetal weight and mean arterial pressure. Observed incidence is given as median with 95% CI (vertical solid lines). Numbers in red are the observed number of women who developed PE and numbers in black below are the total number of women within each estimated risk group. Diagonal line is line of perfect agreement. Overall mean risk is shown by vertical dashed line and overall incidence is shown by horizontal dashed line. Gray boxes represent unaffected pregnancies and red boxes represent pregnancies with PE. AUC, area under the curve.

**Figure 5:**
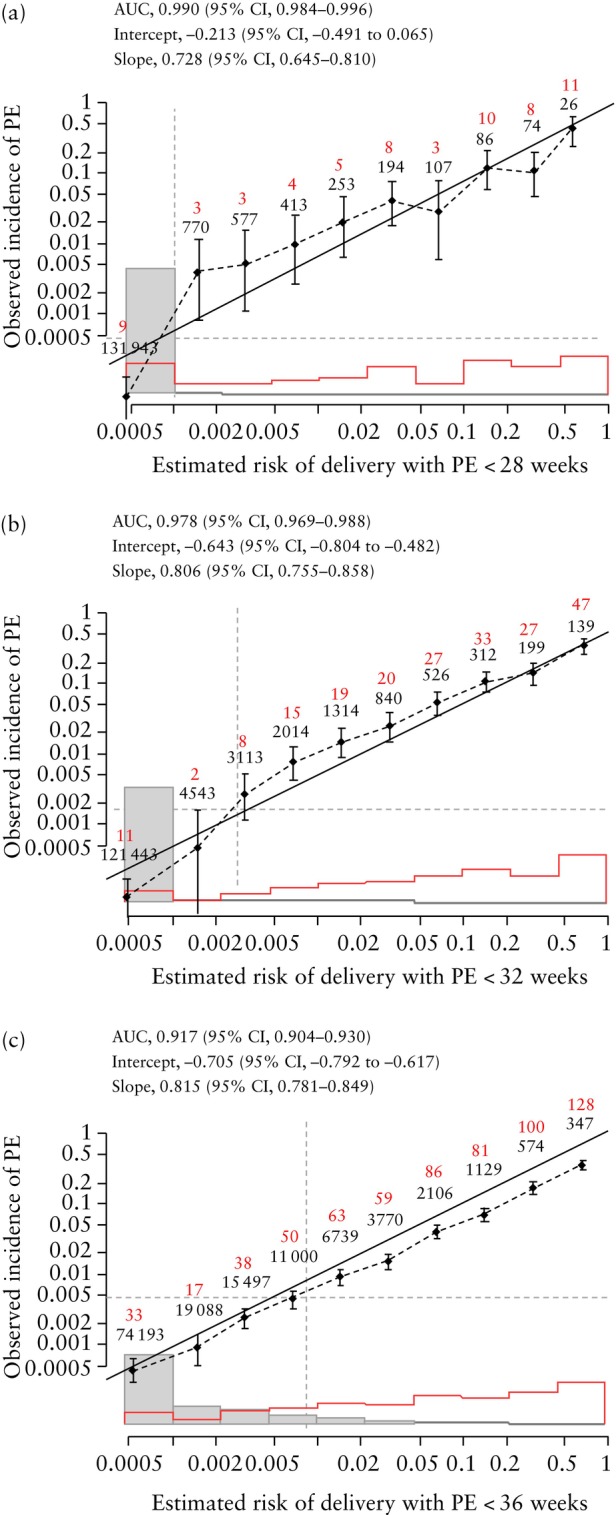
Calibration plots for estimated risk of delivery with pre‐eclampsia (PE) at (a) < 28 weeks, (b) < 32 weeks and (c) < 36 weeks' gestation in screening by maternal risk factors, estimated fetal weight, mean arterial pressure and uterine artery pulsatility index. Observed incidence is given as median with 95% CI (vertical solid lines). Numbers in red are the observed number of women who developed PE and numbers in black below are the total number of women within each estimated risk group. Diagonal line is line of perfect agreement. Overall mean risk is shown by vertical dashed line and overall incidence is shown by horizontal dashed line. Gray boxes represent unaffected pregnancies and red boxes represent pregnancies with PE. AUC, area under the curve.

Calibration plots of the predictive performance of the competing‐risks models for delivery with PE at < 28, < 32 and < 36 weeks' gestation are shown in Figures 4 and 5. The results illustrate good agreement between the estimated risk and observed incidence of PE.

In screening by a combination of maternal risk factors, EFW and MAP, the intercept and slope for prediction of delivery with PE at < 28 weeks' gestation were 0.561 (95% CI, 0.289–0.832) and 0.772 (95% CI, 0.694–0.850); the respective values for delivery with PE at < 32 weeks were 0.084 (95% CI, −0.063 to 0.230) and 0.887 (95% CI, 0.830–0.945), and for delivery with PE at < 36 weeks they were −0.281 (95% CI, −0.361 to −0.201) and 0.932 (95% CI, 0.888–0.976).

In screening using a combination of maternal risk factors, EFW, MAP and UtA‐PI, the intercept and slope for prediction of delivery with PE at < 28 weeks' gestation were −0.213 (95% CI, −0.491 to 0.065) and 0.728 (95% CI, 0.645–0.810); the respective values for delivery with PE at < 32 weeks were −0.643 (95% CI, −0.804 to −0.482) and 0.806 (95% CI, 0.755–0.858), and for delivery with PE at < 36 weeks they were −0.705 (95% CI, −0.792 to −0.617) and 0.815 (95% CI, 0.781–0.849).

## DISCUSSION

### Main findings

This large prospective non‐interventional study of singleton pregnancies attending for a routine mid‐gestation visit and undergoing ultrasound examination has demonstrated an approach for stratification of the population for prediction of subsequent delivery with PE. Effective biomarkers that can be easily recorded in this visit are EFW, MAP and UtA‐PI, and these measurements can be combined with maternal demographic characteristics and elements from the medical history to predict delivery with PE at < 28, < 32 and < 36 weeks' gestation.

The main findings of the study are: first, the incidence of SGA in pregnancies complicated by PE is inversely related to the gestational age at delivery, decreasing from 89.1% in those delivering at < 28 weeks to 22.5% in those delivering at ≥ 36 weeks, and, therefore, EFW *Z*‐score is particularly useful in the prediction of early PE. Similarly, both MAP MoM and UtA‐PI MoM in pregnancies delivering with PE are high, and these values decrease with advancing gestation. Second, effective prediction of delivery with PE at < 28, < 32 and < 36 weeks can be achieved by a combination of maternal risk factors, EFW and MAP. However, the prediction is substantially improved with the addition of UtA‐PI. For example, the SPR necessary to detect 90% of deliveries with PE at < 32 weeks is reduced from 18.3% with only maternal risk factors, EFW and MAP, to 3.8% with the addition of UtA‐PI.

### Comparison with previous studies

Several previous large studies have highlighted the association of preterm PE with SGA^23^, ^24^, ^25^, ^26^. In a previous study in 93 911 women^10^, who are also included in our current study, we highlighted the potential value of EFW as a mid‐trimester biomarker for prediction of subsequent development of preterm PE. In the present study, we have further demonstrated the value of this biomarker in the stratification of pregnancy care.

In a previous study of 7748 singleton pregnancies at mid‐gestation^8^, we reported on the use of the competing‐risks model to combine maternal risk factors with biomarkers for the prediction of subsequent development of PE. A combination of maternal risk factors with MAP and UtA‐PI predicted about 80% of cases of delivery with PE at < 37 weeks' gestation, at a false‐positive rate of 10%^26^. This result is consistent with the findings in this study, in which screening by a combination of maternal risk factors, EFW, MAP and UtA‐PI predicted 80% of cases of delivery with PE at < 36 weeks at a SPR of 11.7%.

The proposal that the mid‐gestation assessment of risk for PE can be used for stratification of the population into risk groups for subsequent care was first made in a study of 16 254 pregnancies^27^. In a subsequent study of 96 678 pregnancies^11^, which are also included in our current study, we demonstrated that the performance of screening by a combination of maternal risk factors and MoM values of MAP and UtA‐PI at 19–24 weeks' gestation for delivery with PE at < 28, < 32 and < 36 weeks is superior to that of screening by fixed cut‐offs of UtA‐PI or by percentile cut‐offs of UtA‐PI. When screening by a combination of maternal risk factors, MAP and UtA‐PI, the SPRs necessary to achieve prediction of 90% of deliveries with PE at < 28, < 32 and < 36 weeks were 2.4%, 6.3%, and 24.1%, which are very similar to the values of 2.6%, 3.8% and 23.6% in this study, in which screening also included EFW. Therefore, EFW is only useful when screening is by maternal risk factors and MAP; once UtA‐PI is included, there is no additional benefit from the inclusion of EFW.

### Implications for clinical practice

Assessment of risk for PE at 20 weeks' gestation is aimed at identification of a high‐risk group that would benefit from close monitoring to minimize adverse perinatal events for those that develop PE by determining the appropriate time and place for delivery. Determination of EFW is an inherent part of the 20‐week scan, and measurement of blood pressure and recording of maternal characteristics and medical history are integral parts of prenatal care, and should therefore not add to current costs. Use of the competing‐risks model to combine maternal risk factors with EFW and MAP can provide accurate prediction of the group of women requiring monitoring of blood pressure starting from 24 weeks' gestation, because they have been identified as being at high risk of delivering with PE at < 28 weeks, those requiring monitoring from 28 weeks, because they have been identified as being at high risk of delivering with PE at < 32 weeks, and those requiring monitoring from 32 weeks, because they have been identified as being at high risk of delivering with PE at < 36 weeks. The proportion of women requiring such close monitoring with the intention of identifying about 90% of women delivering with PE at < 28, < 32 and < 36 weeks' gestation would be 11%, 18% and 39%, respectively.

A more effective approach for assessment of risk for PE would require inclusion of UtA‐PI in the competing‐risks model, which is not currently measured routinely at the mid‐gestation scan. If UtA‐PI was to be included in routine assessment of risk for PE, the DR of 90% of affected pregnancies could be achieved at the considerably lower SPRs of 3%, 4% and 24% for women delivering with PE at < 28, < 32 and < 36 weeks' gestation, respectively. Measurement of UtA‐PI can be carried out within a few minutes by the same sonographer, using the same machines as for the routine second‐trimester scan, at a minimal cost.

The software used for calculation of risk for PE is available freely from the Fetal Medicine Foundation (https://fetalmedicine.org/research/assess/preeclampsia/second‐trimester).

### Strengths and limitations

The strengths of this study are, first, examination of a large population of pregnant women attending for routine care in a gestational‐age range which is used widely for assessment of fetal anatomy and growth; second, recording of data on maternal characteristics and medical history to define the prior risk; third, use of a specific methodology by appropriately trained doctors to measure UtA‐PI and MAP; and fourth, use of Bayes' theorem to combine the prior risk from maternal factors with biomarkers to estimate patient‐specific risks and stratify women into different management groups.

The cut‐offs of risks to define the proportion of the population stratified into those with predicted deliveries with PE at < 28, < 32 and < 36 weeks' gestation and the protocols for such management will inevitably vary according to the characteristics of the study population, local preferences and health‐economic considerations. Future studies are needed to examine whether the implementation of such protocols could improve perinatal outcome.

### Conclusions

All women should be offered assessment of risk for PE at 12 weeks' gestation, to help identify those requiring aspirin prophylaxis to reduce the rate of preterm PE^6^, and at 36 weeks, to determine the optimal timing of birth to reduce the rate of term PE^7^. Assessment of risk for PE at mid‐gestation can be used to identify the subgroups that require additional monitoring at 24–35 weeks, 28–35 weeks and 32–35 weeks' gestation. The best performance of screening, reflected by the SPR necessary to achieve a high DR, is achieved by a combination of maternal risk factors, MAP and UtA‐PI.

## Supporting information


**Table S1** Prediction of delivery with pre‐eclampsia (PE) < 28 weeks' gestation (*n* = 64) in 134 443 pregnancies
**Table S2** Prediction of delivery with pre‐eclampsia (PE) < 32 weeks' gestation (*n* = 209) in 134 443 pregnancies
**Table S3** Prediction of delivery with pre‐eclampsia (PE) < 36 weeks' gestation (*n* = 655) in 134 443 pregnancies

## Data Availability

Research data are not shared.
