# Supplementary material for: Assessment of risk for pre‐eclampsia at mid‐gestation to define subsequent care
Source: Ultrasound Obstet Gynecol. 2025 Apr 18;65(6):694–702. doi: 10.1002/uog.29222 (PMC12127725; doi:10.1002/uog.29222)
Supplement: Supplementary file 1 — Table S1 Prediction of delivery with pre‐eclampsia (PE) < 28 weeks' gestation (n = 64) in 134 443 pregnancies Table S2 Prediction of delivery with pre‐eclampsia (PE) < 32 weeks' gestation (n = 209) in 134 443 pregnancies Table S3 Prediction of delivery with pre‐eclampsia (PE) < 36 weeks' gestation (n = 655) in 134 443 pregnancies [file UOG-65-694-s001.docx]

**Table S1.** Prediction of delivery with preeclampsia (PE) before 28 weeks’ gestation. Total population is 134,443 and total number of cases of PE <28 weeks is 64.

| **Method of screening** | **Risk**  **cut-off**  **(1 in …)** | **Detection** | | **Screen positive** | |
| --- | --- | --- | --- | --- | --- |
|  |  | **n/64** | **DR (95% CI)** | **n/134,443** | **SPR (95% CI)** |
| History | 14000 | 51 | 79.7 (67.8, 88.7) | 48261 | 35.9 (35.6, 36.2) |
|  | 16000 | 54 | 84.4 (73.1, 92.2) | 53814 | 40.0 (39.8, 40.3) |
|  | 20000 | 57 | 89.1 (78.8, 95.5) | 63610 | 47.3 (47.1, 47.6) |
| History + EFW | 6000 | 50 | 78.1 (66.0, 87.5) | 15171 | 11.3 (11.1, 11.5) |
|  | 8500 | 54 | 84.4 (73.1, 92.2) | 19688 | 14.6 (14.5, 14.8) |
|  | 18000 | 57 | 89.1 (78.8, 95.5) | 32255 | 24.0 (23.8, 24.2) |
| History + MAP | 6000 | 51 | 79.7 (67.8, 88.7) | 16140 | 12.0 (11.8, 12.2) |
|  | 12000 | 54 | 84.4 (73.1, 92.2) | 26768 | 19.9 (19.7, 20.1) |
|  | 25000 | 58 | 90.6 (80.7, 96.5) | 41952 | 31.2 (31.0, 31.5) |
| History + UtA-PI | 1500 | 51 | 79.7 (67.8, 88.7) | 5286 | 3.9 ( 3.8, 4.0) |
|  | 2500 | 54 | 84.4 (73.1, 92.2) | 6855 | 5.1 ( 5.0, 5.2) |
|  | 4000 | 57 | 89.1 (78.8, 95.5) | 8713 | 6.5 ( 6.4, 6.6) |
| History + EFW + MAP | 3500 | 50 | 78.1 (66.0, 87.5) | 7262 | 5.4 ( 5.3, 5.5) |
|  | 6000 | 54 | 84.4 (73.1, 92.2) | 10604 | 7.9 ( 7.7, 8.0) |
|  | 12000 | 57 | 89.1 (78.8, 95.5) | 16624 | 12.4 (12.2, 12.5) |
| History + EFW + MAP + UtPI | 300 | 51 | 79.7 (67.8, 88.7) | 1368 | 1.0 ( 0.96, 1.1) |
|  | 600 | 54 | 84.4 (73.1, 92.2) | 1953 | 1.5 ( 1.39, 1.5) |
|  | 2000 | 57 | 89.1 (78.8, 95.5) | 3484 | 2.6 ( 2.5, 2.7) |

**Table S2.** Prediction of delivery with preeclampsia (PE) before 32 weeks’ gestation. Total population is 134,443 and total number of cases of PE <32 weeks is 209.

| **Screening method** | **Risk**  **cut-off**  **(1 in …)** | **Detection** | | **Screen positive** | |
| --- | --- | --- | --- | --- | --- |
|  |  | **n/209** | **DR (95% CI)** | **n/134,443** | **SPR (95% CI)** |
| History | 1500 | 166 | 79.4 (73.3, 84.7) | 49126 | 36.5 (36.3, 36.8) |
|  | 1800 | 179 | 85.6 (80.1, 90.1) | 58004 | 43.1 (42.9, 43.4) |
|  | 2000 | 184 | 88.0 (82.9, 92.1) | 63335 | 47.1 (46.8, 47.4) |
| History + EFW | 1000 | 167 | 79.9 (73.8, 85.1) | 27478 | 20.4 (20.2, 20.7) |
|  | 1500 | 182 | 87.1 (81.8, 91.3) | 38571 | 28.7 (28.5, 28.9) |
|  | 2000 | 189 | 90.4 (85.6, 94.1) | 47541 | 35.4 (35.1, 35.6) |
| History + MAP | 700 | 168 | 80.4 (74.3, 85.5) | 18535 | 13.8 (13.6, 14.0) |
|  | 1000 | 177 | 84.7 (79.1, 89.3) | 25088 | 18.7 (18.5, 18.9) |
|  | 1500 | 188 | 90.0 (85.1, 93.7) | 34447 | 25.6 (25.4, 25.9) |
| History + UtA-PI | 200 | 169 | 80.9 (74.9, 86.0) | 6693 | 5.0 ( 4.9, 5.1) |
|  | 300 | 177 | 84.7 (79.1, 89.3) | 8724 | 6.5 ( 6.4, 6.6) |
|  | 500 | 187 | 89.5 (84.5, 93.3) | 12352 | 9.2 ( 9.0, 9.3) |
| History + EFW + MAP | 500 | 163 | 78.0 (71.8, 83.4) | 11602 | 8.6 ( 8.5, 8.8) |
|  | 700 | 178 | 85.2 (79.6, 89.7) | 15514 | 11.5 (11.4, 11.7) |
|  | 1250 | 189 | 90.4 (85.6, 94.1) | 24577 | 18.3 (18.1, 18.5) |
| History + EFW + MAP  + UtA-PI | 75 | 167 | 79.9 (73.8, 85.1) | 2784 | 2.1 ( 2.0, 2.2) |
|  | 100 | 173 | 82.8 (77.0, 87.6) | 3330 | 2.5 ( 2.4, 2.6) |
|  | 200 | 187 | 89.5 (84.5, 93.3) | 5129 | 3.8 ( 3.7, 3.9) |

**Table S3.** Prediction of delivery with preeclampsia (PE) before 36 weeks’ gestation. Total population is 134,443 and total number of cases of PE <36 weeks is 655.

| **Method of screening** | **Risk**  **cut-off**  **(1 in …)** | **Detection** | | **Screen positive** | |
| --- | --- | --- | --- | --- | --- |
|  |  | **n/655** | **DR (95% CI)** | **n/134,443** | **SPR (95% CI)** |
| History | 250 | 531 | 81.1 (77.9, 84.0) | 53577 | 39.9 (39.6, 40.1) |
|  | 300 | 567 | 86.6 (83.7, 89.1) | 64365 | 47.9 (47.6, 48.1) |
|  | 350 | 592 | 90.4 (87.9, 92.5) | 73277 | 54.5 (54.2, 54.8) |
| History + MAP | 200 | 529 | 80.8 (77.5, 83.7) | 34436 | 25.6 (25.4, 25.9) |
|  | 250 | 554 | 84.6 (81.6, 87.3) | 41629 | 31.0 (30.7, 31.2) |
|  | 400 | 598 | 91.3 (88.9, 93.3) | 58449 | 43.5 (43.2, 43.7) |
| History + EFW | 250 | 538 | 82.1 (79.0, 85.0) | 51617 | 38.4 (38.1, 38.7) |
|  | 300 | 570 | 87.0 (84.2, 89.5) | 61544 | 45.8 (45.5, 46.0) |
|  | 350 | 587 | 89.6 (87.0, 91.8) | 69943 | 52.0 (51.8, 52.3) |
| History + UtA-PI | 100 | 511 | 78.0 (74.6, 81.1) | 17125 | 12.7 (12.6, 12.9) |
|  | 180 | 556 | 84.9 (81.9, 87.5) | 26963 | 20.1 (19.8, 20.3) |
|  | 350 | 590 | 90.1 (87.5, 92.3) | 42269 | 31.4 (31.2, 31.7) |
| History + EFW + MAP | 175 | 523 | 79.8 (76.6, 82.9) | 29492 | 21.9 (21.7, 22.2) |
|  | 250 | 560 | 85.5 (82.6, 88.1) | 40540 | 30.2 (29.9, 30.4) |
|  | 350 | 591 | 90.2 (87.7, 92.4) | 52252 | 38.9 (38.6, 39.1) |
| History + EFW + MAP  + UtA-PI | 100 | 517 | 78.9 (75.6, 82.0) | 14665 | 10.9 (10.7, 11.1) |
|  | 200 | 565 | 86.3 (83.4, 88.8) | 24387 | 18.1 (17.9, 18.4) |
|  | 300 | 590 | 90.1 (87.5, 92.3) | 31732 | 23.6 (23.4, 23.8) |
